# Emerging biomedical imaging applications of tumor-derived extracellular vesicles for premetastatic niche detection: Biological rationale, engineering strategies, and translational challenges

**DOI:** 10.18632/oncotarget.28916

**Published:** 2026-08-28

**Authors:** Omkar Dhaygude, Yun Chen, Yamauchi Suguru, Malcom Brock, Franck Housseau, John Michel

**Affiliations:** ^1^Department of Mechanical Engineering, School of Engineering, Johns Hopkins University, Baltimore, MD 21231, USA; ^2^Department of Surgery/Oncology, School of Medicine, Johns Hopkins University, Baltimore, MD 21231, USA; ^3^Department of Oncology, School of Medicine, Johns Hopkins University, Baltimore, MD 21231, USA; ^4^University of Lille, Inserm, CHU Lille, CNRS, U1366-UMR9020 – CRCLille – Cancer Re-search Center of Lille, F-59000 Lille, France; ^5^Biomedical Engineering Program, Johns Hopkins University, Baltimore, MD 21231, USA

**Keywords:** lung metastases, cancer imaging, theranostic, premetastatic niches, extra-cellular vesicles

## Abstract

Extracellular vesicles (EVs) are a heterogeneous population of membrane-enclosed nanoparticles naturally released by virtually all cell types, including exosomes, microvesicles, and other vesicular subtypes that differ in biogenesis, size, and molecular composition. By transporting proteins, lipids, nucleic acids, and metabolites, EVs mediate local and systemic intercellular communication and play a central role in tumor progression, organ-specific metastasis, and remodeling of distant microenvironments. Recent advances in EV isolation, engineering, and molecular profiling have accelerated interest in their use as nanomedicine platforms for both targeted delivery and cancer diagnostics. In particular, tumor-derived EVs (TEVs) exhibit intrinsic organotropism and participate in the establishment of premetastatic niches, making them promising vehicles for the targeted delivery of imaging probes to metastasis-prone tissues. This review discusses the emerging use of EVs as carriers of contrast and molecular imaging agents to enable visualization of premetastatic niches before overt metastatic lesions become detectable by conventional imaging modalities such as computed tomography. We highlight the biological rationale, engineering strategies, imaging applications, and translational challenges associated with EV-based probe delivery and consider how this approach may improve early detection of metastatic spread, risk stratification, and disease monitoring in oncology.

## INTRODUCTION TO TUMOR-DERIVED EXTRACELLULAR VESICLES (TEVS)

Extracellular vesicles (EVs) are cell-derived, lipid bilayer-delimited particles that do not replicate and are released by virtually all cell types into biofluids and tissue microenvironments [1]. Because EV populations are heterogeneous in size, composition, and biogenesis, current consensus guidelines recommend use of the umbrella term “EV” unless subtype-specific evidence is available [1].

EVs carry complex molecular cargoes, including proteins, lipids, metabolites, DNA, mRNA, microRNA, and other non-coding RNAs, enabling them to function as biologically active mediators of intercellular communication in both homeostatic and pathological settings [2–4]. In cancer, tumor-derived EVs (TEVs) influence multiple components of the tumor microenvironment and the metastatic cascade through the transfer of bioactive molecular cargoes that reprogram fibroblasts, endothelial cells, innate immune cells, and other recipient populations. These interactions promote angiogenesis, extracellular matrix (ECM) remodeling, inflammatory signaling, immune evasion, and therapy resistance, thereby extending the effects of the primary tumor beyond its local site [5–7].

A particularly important function of TEVs is their contribution to premetastatic niche formation in distant organs before overt metastatic colonization becomes clinically detectable [8–10]. Premetastatic niches are permissive microenvironments shaped by reciprocal interactions among tumor-derived factors, local stromal cells, recruited bone marrow-derived cells, vascular compartments, and ECM components [9]. TEVs are increasingly recognized as important mediators of this process because they can deliver organotropic molecular signals that promote vascular leakiness, inflammatory activation, stromal reprogramming, and tissue-specific immune modulation in future metastatic sites. Consistent with these roles, TEV surface molecules, including integrins and other receptors, may influence both tissue tropism and interactions with recipient cells [4]. Commonly used EV-associated markers for characterization include members of the tetraspanin family, such as CD9, CD63, CD81, and CD82, as well as cargo-sorting proteins including ALIX and TSG101 [11–13]. The lung is a major metastatic destination for several malignancies, including sarcoma, breast cancer, melanoma, renal cancer, and head and neck cancers, making pulmonary premetastatic niche biology especially relevant to early metastatic risk assessment [14]. Experimental evidence suggests that TEV surface molecules, particularly integrins, contribute to organ-selective uptake and niche conditioning; notably, exosome integrins α6β4 and α6β1 were associated with lung tropism in a landmark study of organotropic metastasis [15]. While the present review focuses primarily on pulmonary metastasis, the underlying biological principles of TEV-mediated organotropism are likely to extend to other metastatic sites. These findings support the view that TEVs may serve not only as biomarkers of metastatic programming but also as biologically informed vehicles for targeted imaging strategies directed toward metastasis-prone tissues.

Interest in EV-based imaging has grown because EVs combine endogenous biocompatibility with the possibility of loading or associating imaging probes for optical, magnetic, photoacoustic, and nuclear detection [16]. In principle, such platforms could reveal TEV biodistribution, identify tissue sites undergoing early metastatic conditioning, and improve detection of small metastatic lesions. However, this promise should be interpreted cautiously, as most available evidence derives from animal models, and many labelling systems used in proof-of-concept studies, including fluorescent dyes, genetic reporters, quantum dots, and some nanoparticle formulations, remain difficult or inappropriate to translate directly into human imaging [16].

Accordingly, this review examines TEVs as mediators and potential imaging carriers in the context of premetastatic niche detection, with an emphasis on pulmonary metastatic risk. The discussion focuses primarily on two related questions: how imaging probes and labelling strategies can be incorporated into or associated with EVs to track metastatic processes, and which technical, biological, safety, and regulatory barriers are most likely to determine their prospects for clinical translation [1]. Although the contribution of TEVs to premetastatic niche formation is briefly addressed to provide biological context, this topic has been extensively reviewed elsewhere [10, 15, 17, 18]. This review critically evaluates predominantly preclinical TEV-based imaging approaches for premetastatic niche detection through the lens of their translational potential (Figure 1).

**Figure 1:**
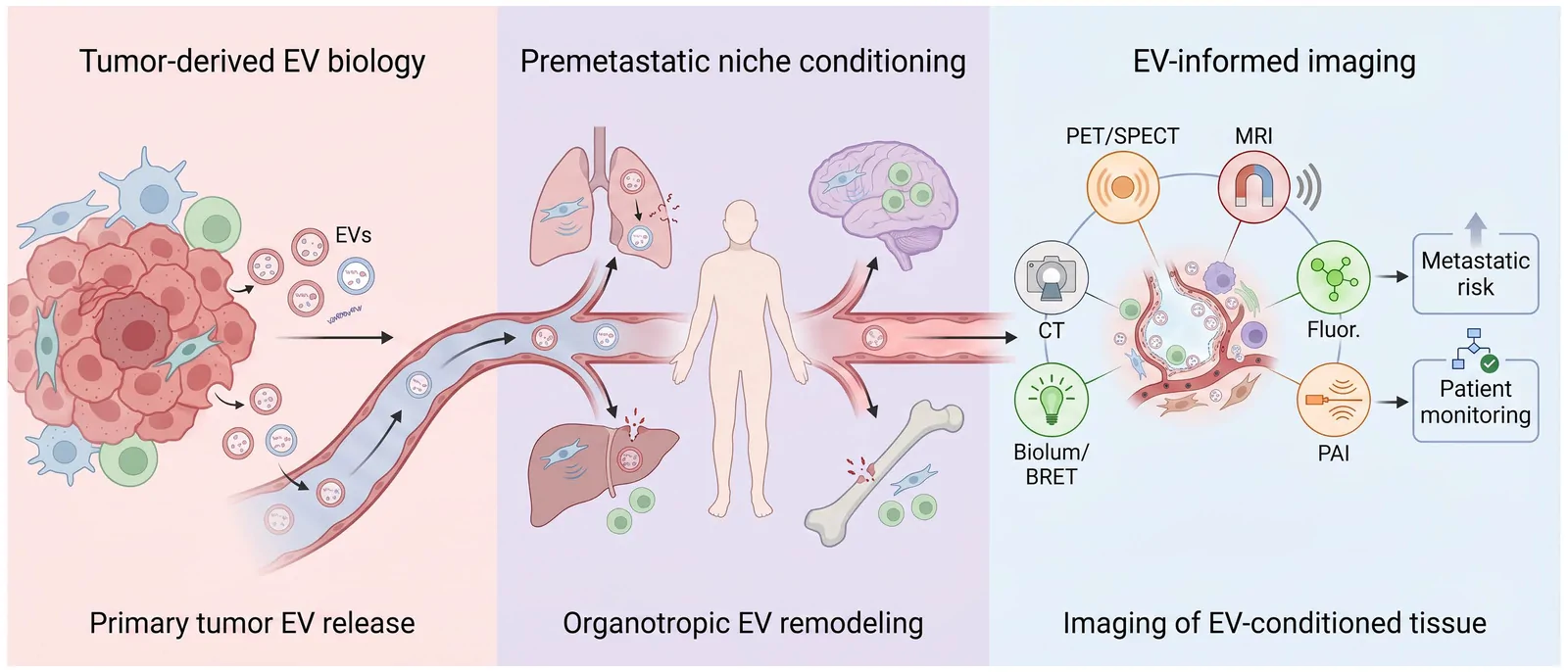
Tumor-derived extracellular vesicles (EVs) contribute to premetastatic niche formation and can be engineered for multimodal imaging to support early metastatic risk assessment and personal-ized therapy.

## ROLE OF TEVS IN THE TUMOR MICROENVIRONMENT (TME)

The TME is not a passive scaffold surrounding malignant cells, but a dynamic environment composed of ECM, vascular networks, fibroblasts, and immune populations that continuously exchange signals with the tumor [19]. TEVs are now recognized as important mediators of this communication because they transfer proteins, lipids, and nucleic acids capable of reprogramming neighboring stromal and immune cells [2–4]. Through these interactions, TEVs help shape local tissue conditions that support tumor growth, invasion, immune escape, and eventual dissemination [17, 20–22]. A schematic overview of the major interactions between TEVs and the cellular and non-cellular components of the TME is presented in Figure 2.

**Figure 2:**
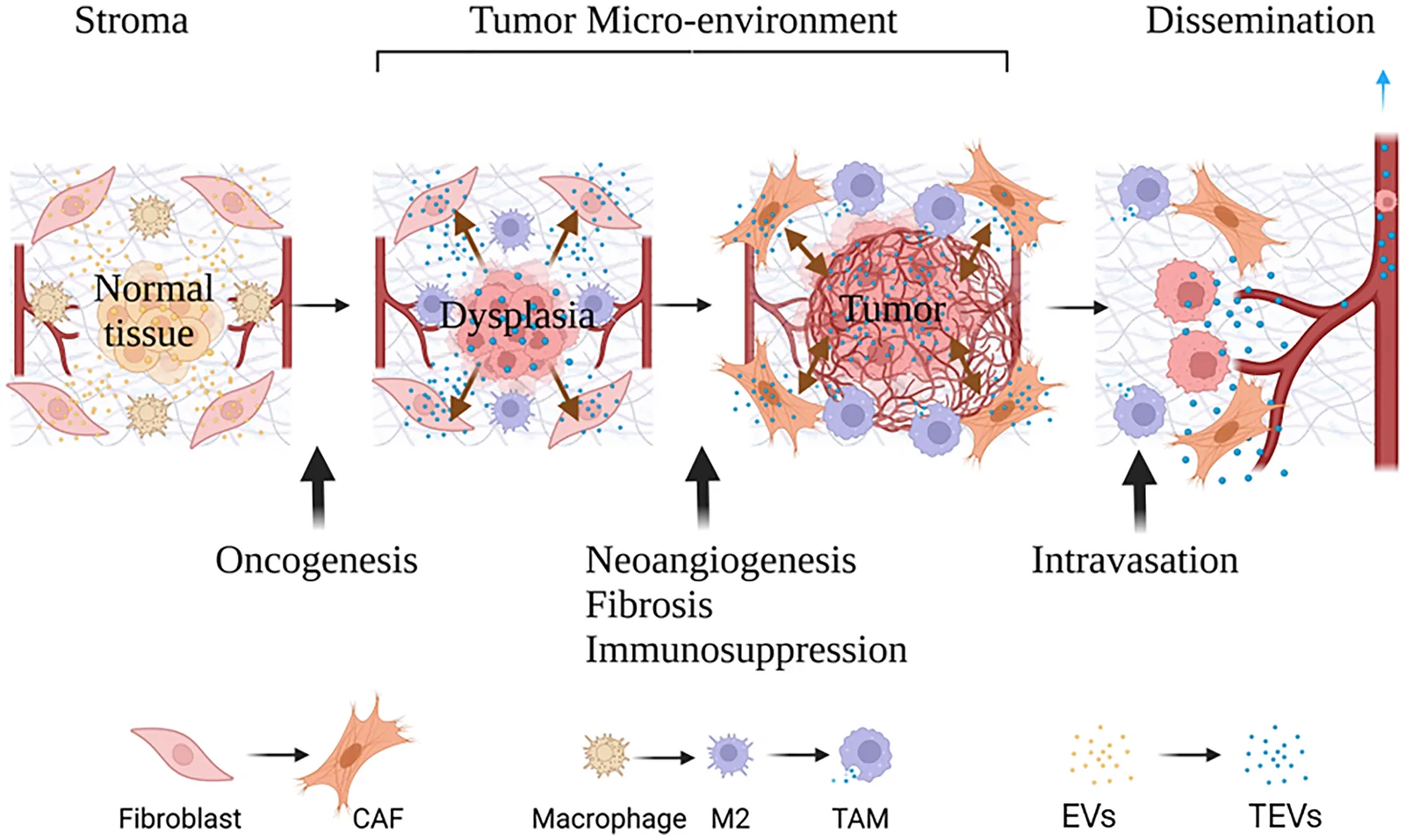
Tumor-derived extracellular vesicles in the tumor microenvironment. Tumor-derived extracellular vesicles (TEVs) mediate bidirectional communication between malignant cells and the major cellular and non-cellular components of the tumor microenvironment. Through interactions with endothelial cells, fibroblasts, immune cells, and extracellular matrix compartments, TEVs contribute to angiogenesis, stromal remodeling, inflammatory signaling, immune evasion, and other tumor-supportive processes that promote local progression and metastatic dissemination. Created in BioRender. Housseau, F. (2025) https://BioRender.com/i11o447.

For the purposes of this review, the most relevant consequence of these local TEV-mediated effects is that they generate biological states (such as endothelial activation, ECM remodeling, inflammatory signaling, immune suppression, and metabolic adaptation) that later become important both for premetastatic niche formation and for the interpretation of TEV-based imaging signals. These effects are mediated by a broad and partially overlapping repertoire of TEV-associated cargoes and surface molecules whose actions depend on tumor type, recipient cell context, and the surrounding tissue environment. Representative EV-associated cargoes implicated in these processes are summarized in Figure 3.

**Figure 3:**
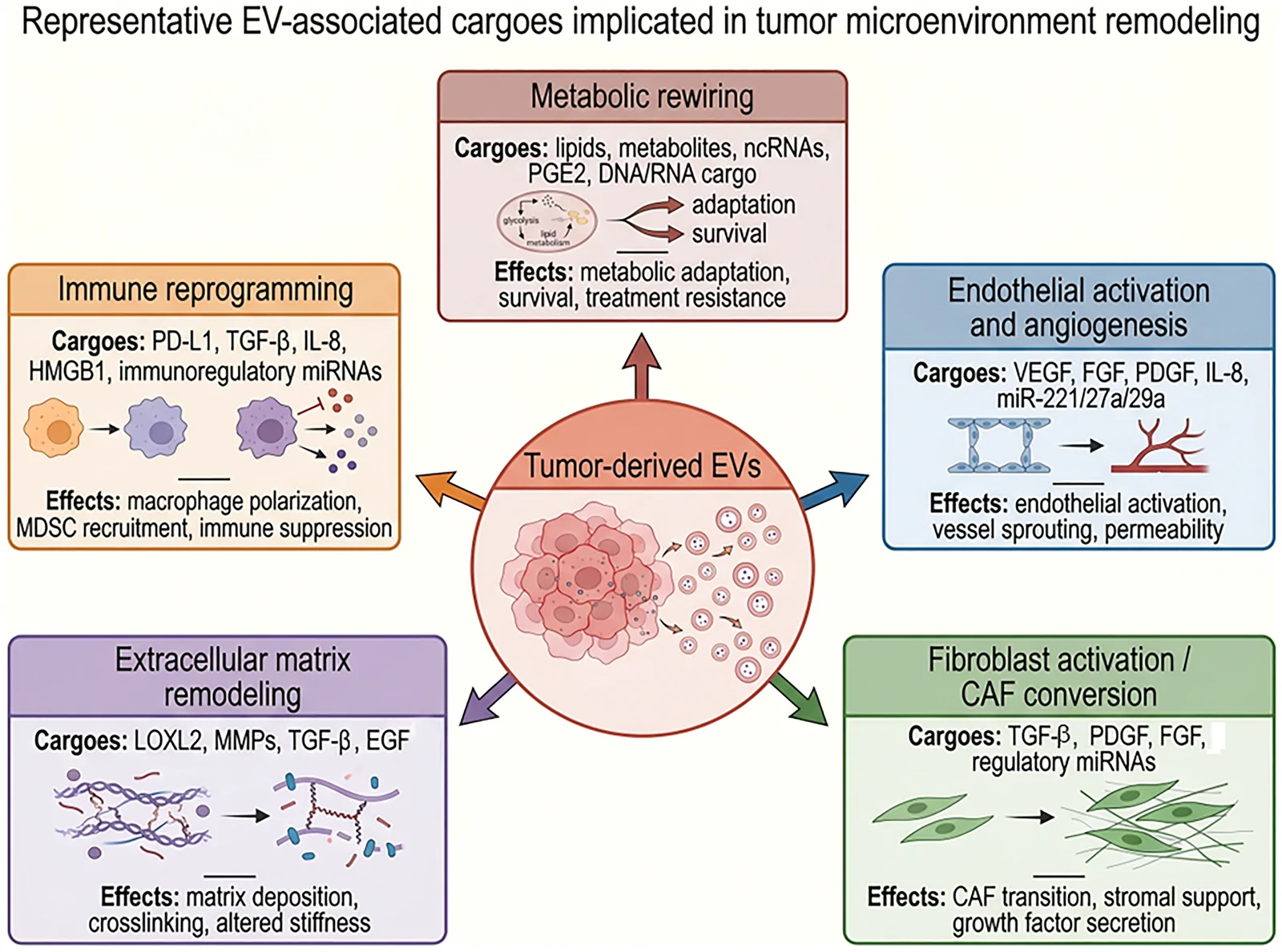
Representative extracellular vesicle-associated cargoes implicated in tumor microenvironment remodeling. Representative TEV-associated cargoes and their reported effects on recipient compartments within the tumor microenvironment are illustrated. These include cargoes associated with endothelial activation and angiogenesis, fibroblast activation and cancer-associated fibroblast conversion, extracellular matrix remodeling, immune reprogramming, and metabolic rewiring. The figure is intended as a conceptual summary of recurrent mechanisms reported across tumor models rather than a comprehensive catalogue of all TEV cargoes.

### Stromal and vascular remodeling in the primary TME

One of the most consistent effects attributed to TEVs in the TME is the reprogramming of endothelial and stromal compartments. TEVs can promote endothelial activation, angiogenic signaling, and vascular leakiness through the transfer of pro-angiogenic mediators, regulatory RNAs, and matrix-remodeling enzymes [23–28]. These effects do not simply increase nutrient supply to the tumor; they also alter tissue architecture and barrier function in ways that facilitate tumor cell intravasation and subsequent dissemination [25–28]. From an imaging standpoint, these vascular changes are especially relevant because they may influence both the access of circulating TEVs to target tissues and the local retention or uptake of TEV-associated probes [23–28].

TEVs also participate in remodeling the ECM and activating fibroblastic populations [25–29]. Fibroblast reprogramming contributes to desmoplasia, altered matrix stiffness, and the production of additional paracrine mediators that reinforce tumor-promoting signaling loops [29]. In parallel, TEV-associated proteases and profibrotic signals can modify matrix composition and organization, thereby creating local microenvironments that are more permissive to invasion [30–34]. These stromal alterations are relevant beyond the primary tumor, because many of the same biological themes (ECM remodeling, endothelial activation, and stromal education) reappear in distant tissues during premetastatic niche formation [9]. In that sense, the TME can be viewed as a local template for the remote tissue conditioning discussed in section 3.

### Immune and inflammatory reprogramming relevant to metastatic spread

TEVs also influence immune surveillance within the TME by acting on macrophages, myeloid-derived suppressor populations, dendritic cells, and lymphocytes [17, 18, 21, 22, 35]. Depending on tumor context and EV cargo, these interactions can enhance inflammatory signaling while paradoxically promoting immune suppression, for example by skewing macrophage polarization, amplifying NF-kB-associated responses, or increasing expression of inhibitory immune checkpoint pathways such as PD-L1. Representative mechanisms include EV-associated PD-L1-mediated inhibition of T-cell activity, macrophage polarization toward tumor-supportive states such as tumor associated macrophages (TAM) or immunosuppressive and profibrotic type 2 macrophages (M2), recruitment or expansion of myeloid-derived suppressor populations, and context-dependent activation of inflammatory pathways such as NF-kB that reinforce immune escape and tumor progression [17, 18, 21, 22, 35]. This dual effect is important biologically because a chronically inflamed but immunosuppressed environment can support tumor progression and later metastatic competence [17, 18, 21, 22]. More recent work has emphasized that these changes should be understood within a broader spatiotemporal framework of premetastatic immune remodeling rather than as isolated local immunosuppressive events [36]. It is also important analytically, because imaging signals associated with EV uptake may reflect immune-cell sequestration or inflammatory remodeling rather than direct tumor cell targeting.

The ability of TEVs to modulate phagocytic interactions and circulation time may further affect their biodistribution [37]. Surface molecules such as CD47 have been implicated in reducing clearance by phagocytic cells, although the extent to which this feature consistently improves delivery or persistence across experimental systems remains to be established [38]. In the context of imaging, these biological variables matter because they can alter background signal, organ uptake, and apparent tropism independently of the intended targeting strategy [39]. As a result, TEV accumulation in a given tissue should not automatically be interpreted as evidence of disease-specific localization; it may also reflect host clearance pathways, inflammatory recruitment, or changes introduced during EV labelling and engineering [16].

## ROLE OF TEVS IN PREMETASTATIC NICHE FORMATION

While the role of TEVs in premetastatic niche biology has been extensively reviewed elsewhere [10, 15, 17, 40], this section summarizes the concepts most relevant to imaging, particularly tissue-level changes that may precede computed tomography (CT)-visible metastases and therefore represent potential targets for molecularly informed imaging approaches [41–45]. The principal mechanisms through which TEVs contribute to distant tissue conditioning and premetastatic niche formation are summarized in Figure 4.

**Figure 4:**
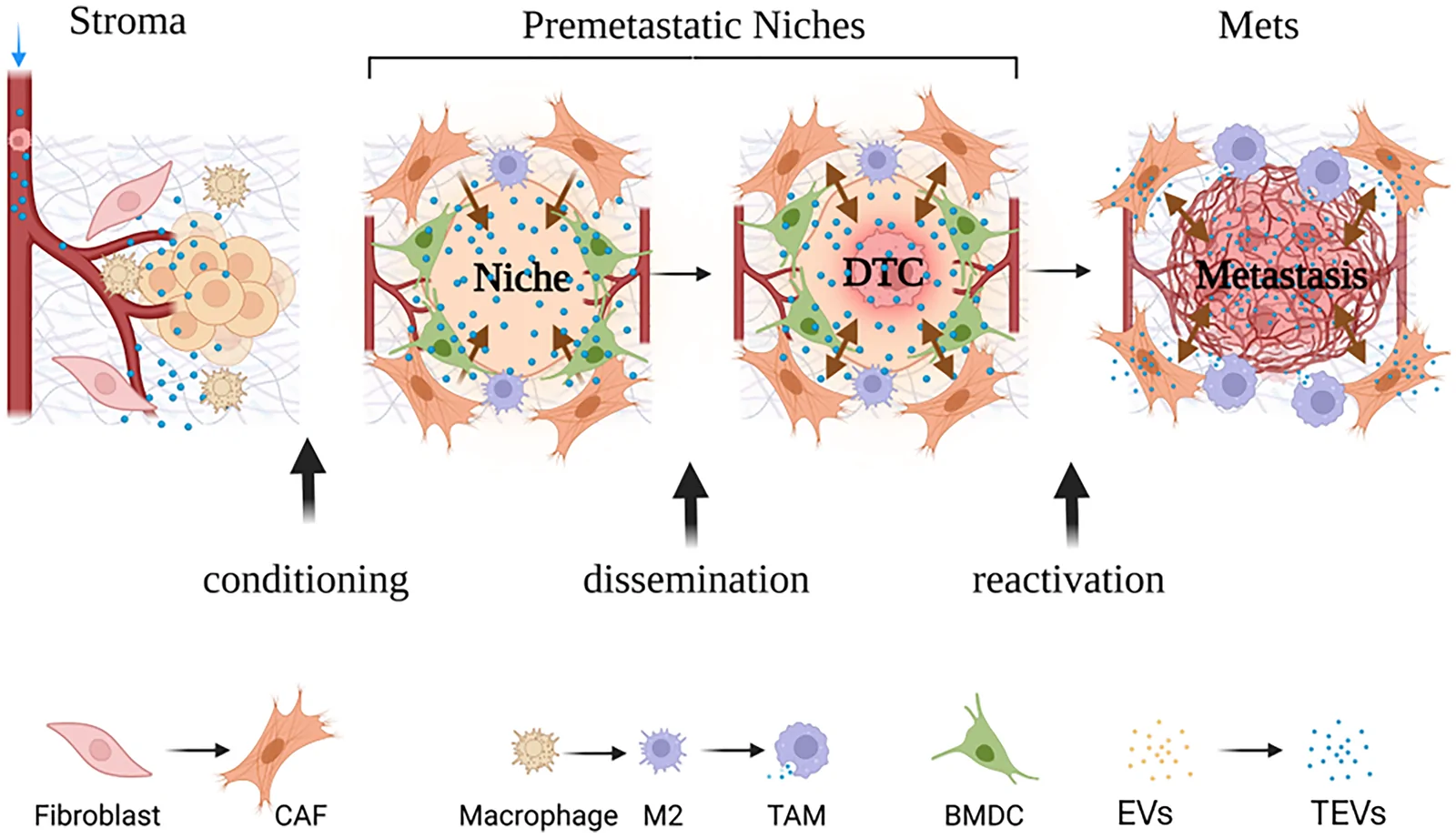
The role of TEVs in the premetastatic niche paradigm. The premetastatic niche arises through tumor-driven reprogramming of distant tissues before overt dissemination and metastatic outgrowth. Tumor-derived extracellular vesicles (TEVs) and soluble mediators prime organ-selective sites by acting on fibroblasts, endothelial cells, and immune populations, thereby promoting angiogenesis, extracellular matrix (ECM) remodelling, immune evasion, and stromal conditioning that support disseminated tumor cell (DTC) survival, dormancy, and eventual reactivation. Created in BioRender. Housseau, F. (2025) https://BioRender.com/j24b061.

### The concept of the premetastatic niche and its translational significance

The concept of the premetastatic niche extends the classical “seed and soil” model by proposing that the soil is actively conditioned before tumor cell arrival [46, 47]. Foundational experimental work demonstrated that distant tissues can be rendered permissive through tumor-driven systemic signaling, thereby supporting subsequent metastatic colonization [46, 47]. More recent frameworks have described recurrent features of the premetastatic niches, including vascular leakiness, lymphangiogenesis, stromal reprogramming, inflammation, immunosuppression, and organotropism [48, 49]. This conceptual framework has been refined by single-cell analyses of the lung premetastatic niche, which identified distinct inflammatory neutrophil, monocyte, and lymphatic endothelial cell states associated with endothelial barrier dysfunction and niche permissiveness [50]. These processes are not independent events; rather, they interact to reshape tissue architecture and local cellular behaviour in ways that favour metastatic competence [48, 49]. The mechanisms through which TEVs contribute to distant tissue conditioning and premetastatic niche formation are summarized in Figure 4.

TEVs are thought to participate in this remodeling by delivering proteins, lipids, nucleic acids, and metabolites to endothelial cells, fibroblasts, epithelial cells, macrophages, neutrophils, and other stromal populations (Figure 3) [10, 15, 17, 36, 40]. The downstream effects include ECM deposition and remodeling, activation of pro-inflammatory signaling pathways, recruitment of bone marrow-derived suppressor populations, and attenuation of anti-tumor immune surveillance [15, 18, 22, 36]. Collectively, these changes may support tumor cell homing and persistence at distant sites [10, 15, 17, 40]. Importantly, the premetastatic niche should not be viewed as a uniform or universal entity. Its composition depends on tumor type, host factors, target organ, and stage of dissemination, which has major implications for imaging because a signal associated with niche formation in one model may not generalize across cancers or organs [48, 49]. This heterogeneity is one reason the premetastatic niche remains both attractive and difficult as an imaging target. Conventional imaging is optimized for structural lesions, whereas the premetastatic niche is defined primarily by molecular and microenvironmental alterations [41–45]. As a result, direct visualization of a bona fide premetastatic niche remains difficult, and much of the current imaging literature more accurately reflects TEV biodistribution, stromal uptake, or detection of very small metastatic deposits rather than definitive niche imaging [45, 51–53]. Nevertheless, if TEV-associated imaging probes can be directed toward tissue changes such as endothelial activation, matrix remodeling, or organ-selective TEV uptake, they may provide earlier and biologically richer information than anatomical imaging alone [45, 53]. This possibility is especially relevant for the lung, where small metastatic foci may remain occult on routine imaging and where metastatic risk stratification remains a major unmet clinical need [54–56].

### Organotropism of TEV trafficking and relevance to pulmonary premetastatic niche formation

A central reason TEVs are attractive as imaging carriers is that they do not distribute randomly [15]. In addition to being passively filtered through highly perfused organs such as the liver, spleen, and lungs, TEVs may display organotropism through surface adhesion molecules and receptor-ligand interactions inherited from their cells of origin [10, 15, 17, 57–61]. A landmark study showed that TEV integrin profiles are associated with organ-specific metastatic patterns, with integrins α6β4 and α6β1 linked to lung tropism and integrin αvβ5 associated with liver tropism [15]. This observation has become an important biological basis for the hypothesis that TEVs could be used not only as biomarkers of metastatic propensity but also as vehicles to target imaging probes toward metastasis-prone tissues [15, 17, 60, 61].

In the context of pulmonary metastasis, TEVs may contribute to early niche formation by interacting with lung endothelial cells, fibroblasts, epithelial populations, and recruited myeloid cells [54, 55, 59]. Emerging evidence indicates that lung-resident stromal programs, including fibroblast activation states, may contribute directly to organ-specific niche permissiveness rather than merely reflecting passive responses to circulating tumor-derived factors [62]. These interactions can promote vascular permeability, inflammatory activation, ECM remodeling, and immune modulation, thereby generating a lung microenvironment that is more permissive to subsequent tumor cell seeding [15, 40, 54, 55, 59]. Experimental studies in lung-tropic models have supported the idea that TEV-mediated niche conditioning occurs before overt metastatic outgrowth, although the degree to which these findings can be generalized across tumor types remains uncertain [15, 54, 55, 59]. Recent work has further suggested that fibroblast STAT3 activation may represent a lung-specific premetastatic stromal program, reinforcing the concept that pulmonary niche formation cannot be reduced to passive TEV trapping in a highly perfused organ [62]. Moreover, biodistribution studies must be interpreted cautiously, because accumulation in the lung may reflect both biological tropism and physical trapping or route-dependent delivery effects [10, 63, 64].

This distinction is especially important for imaging studies. Apparent signal enrichment in the lung does not necessarily indicate selective visualization of a premetastatic niche; it may also reflect nonspecific uptake, reticuloendothelial sequestration, or altered circulation kinetics introduced by labelling procedures [51–53, 63, 64]. For this reason, studies of TEV-based imaging should ideally distinguish among native organotropism, probe-driven redistribution, and true niche-associated accumulation [51–53]. They should also address whether the imaging signal corresponds to a biologically meaningful event, such as stromal activation or endothelial remodeling, rather than simply particle localization [41–45].

Taken together, current evidence supports the idea that TEV trafficking and organotropism provide a biologically plausible basis for imaging metastasis-prone tissues, including the lung [15, 54, 55, 60, 61]. However, this evidence remains predominantly preclinical, and the field still lacks standardized methods to distinguish TEVs from background EV populations, validate niche-specific signal sources, and determine which imaging platforms are realistic for clinical translation. These limitations motivate the next section, which examines how EVs are labelled, engineered, and imaged *in vivo* and which strategies currently appear most promising from a translational perspective [56, 63, 64].

## TRACKING TEVS *IN VIVO* TO DETECT EARLY METASTATIC RISK

Researchers have investigated various imaging strategies to track EVs *in vivo*, with positron emission tomography (PET), magnetic resonance imaging (MRI), and computed tomography (CT) currently offering the clearest translational potential because they provide deep-tissue or whole-body readouts [16, 39]. By contrast, fluorescence- and bioluminescence-based methods have been especially valuable in preclinical studies of EV biodistribution, uptake, and organotropism, but their clinical relevance remains constrained by shallow tissue penetration, label instability, and reliance on engineered reporter systems (Table 1) [56, 63, 64]. Importantly, most existing approaches visualize labelled TEV-associated signal rather than the premetastatic niche itself and should therefore be interpreted as proxy readouts of niche conditioning rather than validated tools for direct niche imaging [65, 66].

**Table 1:** Characteristics of imaging approaches

Modality	Spatial resolution	Sensitivity	Imaging window	Tissue penetration
NIR fluorescence	Moderate (0.1–1 mm superficially; poorer at depth)	High (nM-pM)	Mins to hrs	Mms to 1–2 cm
Bioluminescence/BRET	Moderate (mm-scale *in vivo*)	Very high (fM-pM)	Mins to hrs	Limited-moderate, mainly small animals
Photoacoustic imaging	High (~50–300 μm)	Moderate (nM-μM)	Mins to hrs	Mms to several cms
PET	Low-moderate (1–5 mm)	Very high (pM-nM)	Hrs to Ds	Whole-body
SPECT	Low-moderate (1–2 mm preclinical; 7–15 mm clinical)	High (nM-pM)	Hrs to Ds	Whole-body
CT	Very high (~10–200 μm preclinical; 0.1–1 mm clinical)	Low (μM-mM)	Mins to hrs	Whole-body
MRI	High (~50–200 μm preclinical; 0.5–1 mm clinical)	Low (μM-mM)	Hrs to Ds	Whole-body

Values are approximate practical ranges and vary with labelling chemistry, instrument settings, acquisition protocol, and imaging depth. For optical methods, apparent spatial resolution worsens with increasing tissue depth. Abbreviations: Ds: days; hrs: hours; Mms: millimeters; Mins: Minutes.

In recent years, TEVs have emerged as valuable tools for studying the biodistribution of metastatic processes and for probing how distant tissues may be conditioned before overt metastatic colonization. However, many published strategies rely on membrane dyes, radiolabels, nanoparticle loading, or genetically encoded reporters, meaning that the observed signal often reflects a modified TEV platform rather than an unperturbed native EV population (Figure 5) [65]. Accordingly, the major imaging modalities currently used to study EV trafficking should be assessed not only for sensitivity and image quality, but also for biological fidelity, tumor specificity, and translational feasibility. [56, 63, 64]. Table 2 summarizes the principal imaging modalities currently used for EV tracking and ranks them according to their typical translational relevance. The table emphasizes the distinction between clinically established whole-body imaging approaches, such as PET/SPECT and MRI, and predominantly preclinical optical, nanotechnology-enabled, and molecular biology-based methods that currently serve mainly mechanistic or proof-of-concept roles. A detailed study-level inventory of representative EV imaging reports, including model systems, probe classes, validation methods, *in vivo* imaging platforms, and probe-association strategies, is provided in Supplementary Table 1.

**Figure 5:**
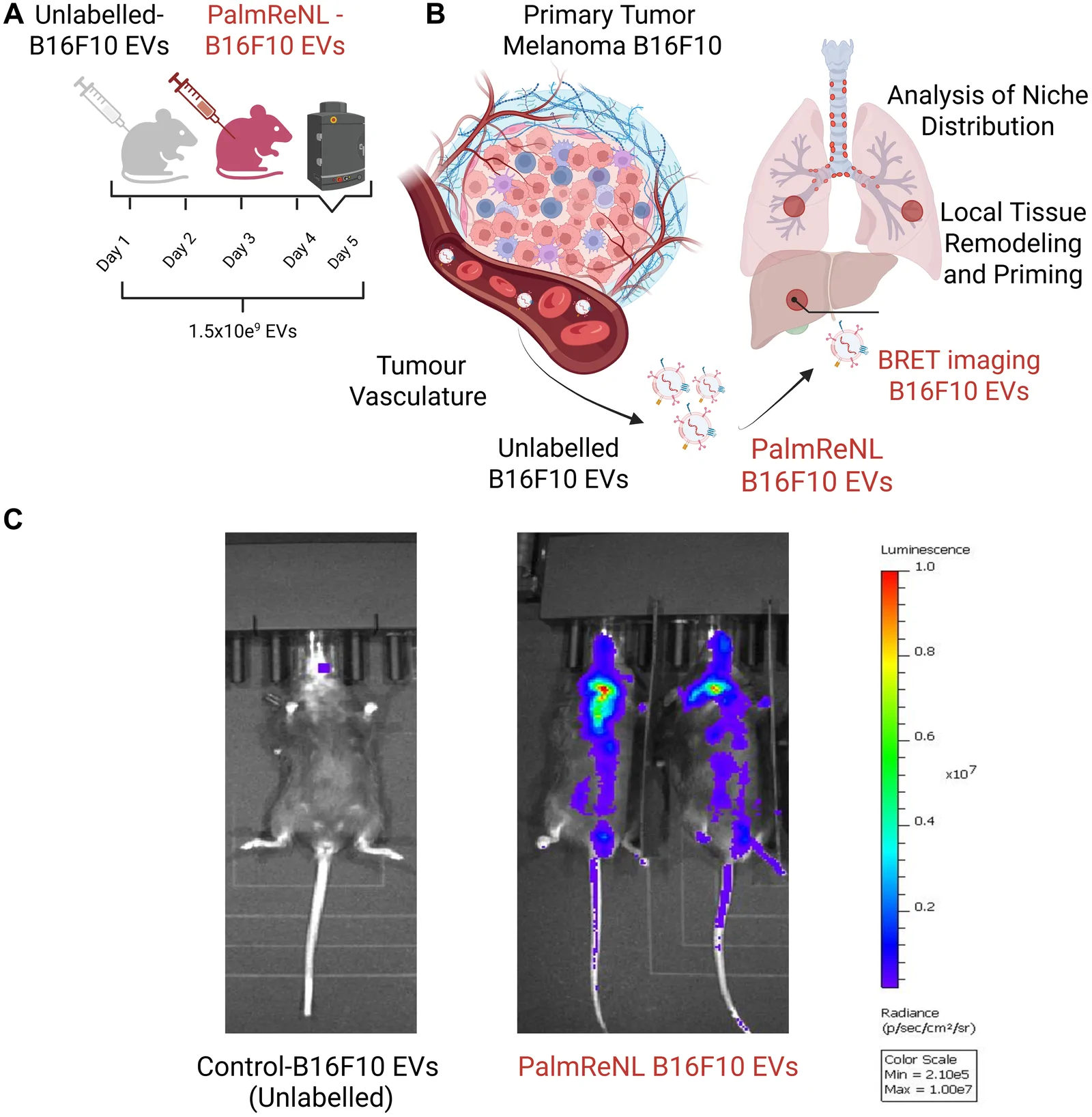
Bioluminescence-based tracking of tumor-derived extracellular vesicles (EVs) in a preclinical vascular access button mouse model to image the pre-metastatic niche. (**A**) Experimental workflow illustrating the intravenous administration of unlabeled B16F10 EVs (control in black) or PalmReNL-labeled B16F10 EVs (in red) over five consecutive days (1.5 × 10^9^ EVs per injection), followed by longitudinal *in vivo* bioluminescence imaging via IVIS. (**B**) Schematic depicting the release of EVs from the primary tumour into the circulation and their accumulation within the lung and liver pre-metastatic niche. PalmReNL-labeled EVs will enable for BRET-based bioluminescence imaging to evaluate EV biodistribution, tissue remodeling, and niche priming *in vivo*. (**C**) Representative *in vivo* bioluminescence images comparing mice receiving unlabeled B16F10 derived EVs (left text in black) with PalmReNL-labeled B16F10 EVs (right text in red), demonstrating robust whole-body luminescence following systemic administration of labeled EVs as described in (A). This example highlights the utility of tumour derived EVs for non-invasive longitudinal tracking of pre-metastatic niches in living animals. Unpublished work; Created in BioRender. Housseau, F. (2026) https://BioRender.com/nvz5y23.

**Table 2:** Key features of imaging modalities used for EVs tracking, ranked by translational relevance

Modality class	EV labelling/tracking strategy	Application	Strengths	Limitations	Translational level
PET/SPECT	Radiolabelling with isotopes technetium, iodine, copper, indium, or zirconium derivatives	*in vivo* biodistribution, organ tropism, quantitative EV tracking	Very high sensitivity; deep-tissue imaging; whole-body readout; strongest current route toward clinical EV biodistribution studies	free tracer, radiolabel instability, hepatic or splenic sequestration, or contrast persistence may not reflect intact EVs	**High**
MRI	Magnetic loading with iron oxide nanoparticles or reporter systems (ferritin-based constructs)	Deep-tissue localization with anatomical co-registration	Excellent soft-tissue contrast; no ionizing radiation; strong anatomical information	Lower molecular sensitivity than PET/SPECT; Alteration of EV behavior; contrast persistence may not reflect intact EVs	**High to Moderate**
CT	Incorporation of radiodense materials (gold nanoparticles or related dense contrast agents)	Anatomical localization and multimodal EV tracking	Widely available; high anatomical resolution; useful as a complementary modality	Low molecular sensitivity; dense exogenous contrast loading; limited value as a stand-alone EV tracking approach	**Moderate (complementary modality)**
Photoacoustic imaging	Loading with optically absorbing dyes or nanoparticles	Deeper-tissue EV tracking and theranostic studies	Better depth than conventional fluorescence; good contrast in preclinical models	Usually requires non-native absorbing agents; limited standardization; uncertain clinical translation	**Preclinical**
Fluorescence imaging	Membrane dyes, NIR fluorophores, fluorescent proteins, or fluorescent nanoparticle-associated probes	Sensitive EV biodistribution, uptake, and cellular localization	Accessible; versatile; useful for mechanistic studies and small-animal imaging	Shallow tissue penetration, autofluorescence, signal scattering, and label dissociation	**Preclinical**
Bioluminescence/BRET	Luciferase-tagged EV proteins, reporter-engineered donor cells, and substrate-dependent bioluminescent readouts	Longitudinal tracking of EV secretion, biodistribution, and uptake	Very high sensitivity and low background	Engineered reporters and exogenous substrates; limited anatomical information	**Preclinical**
Nanotechnology-enhanced EV tracking	Quantum dots, carbon dots, magnetic-fluorescent nanoarchitectures, metal nanoclusters, aptamer-linked nanoparticles, or other multimodal nanoprobes	Signal amplification, multiplexed detection, and proof-of-concept multimodal tracking	Strong tunability and high signal output; enables multimodal probe design	Potential toxicity, non-native cargo effects, poor standardization, and limited regulatory readiness	**Experimental/preclinical**
Molecular biology-based tracking systems	Cre-loxP, CRISPR-based reporters, CD63/CD9/CD81 fusion constructs, and other genetically encoded lineage-tracing approaches	Mechanistic studies of EV origin, cargo transfer, and recipient-cell specificity	High biological specificity; strong mechanistic insight	Requires engineered donor and/or recipient systems; not suitable for direct clinical imaging	**Mechanistic research only**

EV-associated signal in the cited literature often reflects labelled, engineered, or probe-loaded vesicles rather than direct visualization of unmodified native tumor-derived EVs. Translational level refers to the imaging platform and not to clinical validation of EV-specific applications.

### Extracellular vesicle-based optical imaging

Optical imaging approaches, including fluorescence and bioluminescence methods, have been especially valuable in preclinical studies because they enable sensitive visualization of EV biodistribution, uptake, and intercellular transfer without requiring ionizing radiation. These approaches are particularly useful for mechanistic studies of TEV trafficking during early metastatic dissemination, although their applicability to human *in vivo* imaging remains limited [56, 63, 64]. In general, optical methods offer high experimental sensitivity but modest translational potential because tissue scattering, shallow penetration, and the need for engineered reporters or non-clinical probes constrain their use outside small-animal systems [66].

#### Fluorescence confocal imaging

Recent advances in fluorescent labelling have improved the visualization of TEVs *in vivo*, especially through the development of biocompatible and photostable dyes that emit in the near-infrared (NIR) and NIR-II ranges. These approaches are attractive in preclinical settings because they avoid ionizing radiation and can provide sensitive readouts of TEV localization in superficial tissues and small-animal models. However, their performance remains depth-dependent, and signal localization may be confounded by tissue scattering, background fluorescence, and label dissociation from the vesicle membrane [65, 66].

##### Lipophilic dyes

Traditional fluorescence tracking approaches often use lipophilic dyes, but these can suffer from high background signal, weak membrane association, fluorophore release, and nonspecific transfer to non-EV membranes. Nevertheless, such methods laid the groundwork for subsequent optical tracking platforms such as BODIPY-based dyes, which provide brighter and more stable labelling with less apparent disruption of EV structure. [67]. Similarly, Sulfo-cyanine 7.5 has been used for IVIS monitoring of EV biodistribution while reducing autofluorescence because of its NIR emission and water solubility [67]. Despite these advantages, lipophilic labelling remains primarily an experimental strategy, and its translational value is limited by off-target membrane labelling, uncertain signal fidelity after EV degradation, and the impracticality of whole-body optical imaging in humans [65].

##### Quantum dots and polymer dots

Quantum dots are semiconductor nanocrystals with strong fluorescence intensity, photostability, and tunable emission spectra, allowing multiplexed imaging applications [68, 69]. They can be associated with EV membranes or incorporated into the EV lumen through electroporation [70]. Water-soluble perovskite quantum dot systems and dual-color NIR-II quantum dot labelling strategies have been used to track distinct EV populations and to link biodistribution patterns with differential surface protein composition [68, 71]. Liu et al. synthesized ultrasmall Ag_2_Se (1020 nm emission) and Ag_2_Te (1550 nm emission) NIR-II quantum dots and used them to label distinct populations of TEVs from B16F10 melanoma cells, enabling dual-color *in vivo* fluorescence imaging, revealing that the differential organotropism of TEVs is associated with their surface protein profiles [68]. These approaches may offer advantages over earlier cadmium-based quantum dots, but their translational potential remains limited [72]. These systems have substantial limitations, including low loading efficiency, the need for extensive purification to remove unbound nanoparticles, and persistent concerns regarding toxicity and regulatory feasibility, especially when heavy-metal-containing probes are considered for eventual human use. These issues make quantum-dot-based EV imaging informative experimentally but unlikely to provide a near-term clinical route for premetastatic niche detection [66].

##### Templated gold and silver nanoclusters

EVs loaded with gold or silver nanoclusters offer strong fluorescence in the NIR and NIR-II range, improved photostability, and the possibility of multimodal imaging [73]. In some tumor-bearing models, precursor ionic solutions have been used to generate inorganic nanoclusters selectively within neoplastic cells, after which the released TEVs also carried the nanocluster signal. Co-administration of Au and Fe precursor systems has further enabled multimodal fluorescence, CT, and MRI applications. However, this strategy depends on local oxidative conditions and engineered loading environments that may not be replicated in premetastatic tissues, making the approach biologically conditional and potentially difficult to generalize to clinically relevant premetastatic niche imaging [74].

##### Cre/loxP- and CRISPR-based fluorescence tracking

Genetic lineage-tracing systems have provided some of the most elegant demonstrations of EV-mediated cargo transfer. Zomer et al. used the Cre-loxP system to visualize EV transfer between donor and recipient cells through a color-switch reporter, enabling highly specific *ex vivo* and *in vivo* identification of recipient cells that had received EV cargo [75]. Related CRISPR-based systems such as CROSS-FIRE were later developed to trace EV-mediated RNA transfer by inducing a reporter switch after sgRNA delivery to recipient cells [76]. These approaches are powerful because they improve cell-of-origin resolution and distinguish true cargo transfer from passive label accumulation. However, because they require genetic engineering of donor and/or recipient cells, they are best viewed as mechanistic experimental tools rather than clinically deployable strategies for human TEV imaging [65, 66].

#### Bioluminescence imaging

Bioluminescence imaging has emerged as a highly sensitive method for tracking EVs *in vivo*, particularly in small-animal models where low background signal enhances detection [16]. Although bioluminescence generally offers poorer anatomical resolution than fluorescence or tomographic methods, it is well suited to longitudinal studies of EV pharmacokinetics and organ distribution. BRET-based systems such as PalmGRET have further expanded these capabilities by combining bioluminescent and fluorescent readouts in a single platform. Wu et al. used PalmGRET to track TEVs derived from hepatocellular carcinoma to demonstrate a predominant signal accumulation in the lungs and liver, with lower signal in the kidneys, heart, and brain [77]. They used a spontaneously metastatic syngeneic orthotopic hepatocellular carcinoma model in immunocompetent mice [78]. Similar reporter systems, including PalmReNL and luciferase-tagged CD63, CD9, or CD81 constructs, have also been used to study EV secretion, trafficking, and uptake [79–81]. In parallel, mRNA-loaded EVs encoding light-emitting reporters such as Antares2 have been used to generate strong *in vivo* bioluminescent signal after systemic administration [82]. Taken together, these methods provide highly sensitive tools for studying EV kinetics and biodistribution *in vivo*. However, they require engineered reporters and exogenous substrates such as luciferin or furimazine analogues and therefore have limited direct clinical applicability. As with fluorescence approaches, bioluminescent readouts provide strong experimental evidence for TEV trafficking but do not yet constitute clinically realistic methods for direct premetastatic niche imaging [66].

### Photoacoustic imaging

Photoacoustic imaging offers an intermediate strategy between optical and cross-sectional imaging by converting absorbed pulsed light into ultrasound signals, thereby improving tissue penetration relative to conventional fluorescence imaging [83]. In EV applications, this usually requires loading vesicles with optically absorbing dyes or nanoparticles that can generate robust photoacoustic contrast after laser excitation. This feature makes photoacoustic imaging attractive for studies that seek deeper tissue interrogation while retaining some of the sensitivity and molecular contrast advantages of optical methods [83]. Hill et al. demonstrated the use of multispectral optoacoustic tomography (MSOT) with Prussian blue nanoparticle-loaded TEVs in a murine glioblastoma model, showing tumor accumulation and photoacoustic detectability together with a photothermal therapeutic effect [84]. Cao et al. used indocyanine green, an FDA-approved NIR dye, to improve TEV photostability and permit photoacoustic imaging in tumor-bearing mice [85]. Jang et al. similarly loaded melanoma-derived EVs with chlorin e6, enabling photoacoustic imaging together with laser-induced tumor cytotoxicity *in vivo* [86]. These studies illustrate the theranostic (detection+treatment) flexibility of photoacoustic EV platforms. However, they also highlight an important limitation: the EVs must usually be modified with non-native absorbing agents, and those modifications may alter vesicle composition, biodistribution, or uptake. Thus, while photoacoustic imaging offers a useful compromise between depth and contrast in preclinical studies, it remains an engineered strategy whose translational feasibility will depend on improved safety, standardization, and preservation of EV biological fidelity [66].

### Positron Emission Tomography (PET), Single-Photon Emission Computed Tomography (SPECT), Computed Tomography (CT), and Magnetic Resonance Imaging (MRI)-based EV imaging

Among the modalities currently used for *in vivo* EV imaging, nuclear imaging and clinically established cross-sectional modalities offer the clearest route toward eventual human translation because they provide whole-body or deep-tissue readouts. In practice, EVs are typically rendered detectable by radiolabelling for PET or SPECT, by magnetic loading or reporter expression for MRI, or by incorporation of high-density materials for CT [16]. These approaches are especially relevant when the goal is to study systemic biodistribution, organ tropism, and the possibility of detecting early metastatic conditioning in internal organs.

#### PET/SPECT imaging

Radiolabelled EVs have been used to visualize biodistribution and organ accumulation *in vivo*, and several studies have shown that rapid labelling with radionuclides such as iodine isotopes can achieve high labelling efficiency without obvious disruption of EV properties [87]. PET imaging using EVs labelled with copper-based tracers has also demonstrated high sensitivity for small metastatic lesions in preclinical lung metastasis models. Because PET and SPECT are highly sensitive and clinically established whole-body modalities, they represent some of the most plausible routes for future EV biodistribution studies in humans. However, interpretation remains challenging because signal may reflect free tracer, label instability, hepatic and splenic sequestration, or persistence of the radiolabel after EV degradation rather than intact vesicle localization alone [87].

#### CT imaging

CT-based EV tracking generally requires incorporation of dense contrast agents such as gold nanoparticles to generate detectable signal [52]. Gold-labelled TEVs have been used to track biodistribution and metastatic localization while preserving TEV morphology and uptake in some preclinical systems. However, CT has relatively poor molecular sensitivity compared with nuclear imaging, and at least one metastatic melanoma model showed insufficient resolution for detecting small pulmonary lesions [52]. Thus, CT is best viewed as a complementary modality that may provide strong anatomical context within multimodal imaging workflows rather than a stand-alone method for sensitive EV-based premetastatic niche detection.

#### MRI

MRI provides excellent soft-tissue contrast and deep-tissue imaging without ionizing radiation, making it an appealing modality for EV tracking [88]. EV localization has been studied using iron oxide nanoparticles and ferritin-based reporter systems, enabling non-invasive visualization in mouse models [45, 89]. Hu et al. further showed that melanoma-derived EVs loaded with superparamagnetic iron oxide nanoparticles (SPIONs) could be detected in lymph nodes while retaining some homing properties of the native vesicles [45]. Nonetheless, MRI remains less sensitive than PET or SPECT for molecular tracking, and its reliance on magnetic loading raises concerns about altered EV behaviour, susceptibility-related false-positive signal, and uncertain correspondence between contrast persistence and intact EV presence [66]. Taken together, these modalities offer the strongest translational framework for future EV imaging because they can access deep tissues and, in some cases, provide whole-body quantitative readouts. Even so, current evidence remains largely preclinical, and none of these platforms have yet established robust, clinically validated imaging of the premetastatic niche through endogenous tumor-derived EV tracking alone.

### Major translational limitations of current EV imaging strategies

Despite substantial technological progress, several major limitations continue to impede the clinical translation of EV imaging (Table 2). These include imperfect fidelity of the imaging label, insufficient specificity for TEVs, perturbation of EV biology during loading or engineering, and the lack of reproducible large-scale clinical-grade EV production [83, 90]. Additionally, the key limitations associated to the imaging modalities also apply to the EV detection [91–95].

A first major challenge is *label fidelity*. Fluorescent dyes, radiolabels, and nanoparticle-based contrast systems may dissociate from the vesicles, persist after EV degradation, or redistribute independently of intact EV trafficking. As a result, the measured signal may not always correspond to the true localization of biologically active EVs [65]. This issue is particularly important when attempting to infer subtle premetastatic niche conditioning from low-intensity or temporally persistent signals.

A second limitation is *tumor specificity*. Most current imaging methods detect labelled vesicles or label-associated signal, but do not reliably distinguish TEVs from EVs released by stromal, immune, or other host tissues. Experimental systems such as Cre-loxP can improve specificity by tracing donor-recipient transfer, but such strategies require engineered models and are not currently applicable to routine human imaging [96]. Therefore, the ability to identify TEVs *in vivo* remains a major unresolved challenge for the field.

A third limitation is *biological perturbation*. Many imaging approaches require membrane dyes, radionuclides, SPIONs, gold nanoparticles, or other exogenous materials that may alter EV size, membrane composition, uptake, circulation time, or organ tropism. This raises a fundamental interpretive problem: a signal-rich engineered EV may not fully recapitulate the behaviour of the native vesicle population that originally participates in premetastatic niche formation [66].

Finally, *clinical manufacturability and regulatory feasibility* remain substantial barriers. Discussions of EV imaging as a translational platform must consider low EV yield, heterogeneity across preparations, lack of reproducibility among isolation methods, and the difficulty of generating GMP-compatible clinical-grade EV formulations at scale [1, 92, 97]. In addition, safety concerns remain unresolved for several labelling strategies, including radioactive tracers, heavy-metal-based contrast agents, and genetically engineered reporter systems [16, 65, 66]. These constraints are especially important when considering repeated administration for biodistribution studies or early-risk imaging in patients rather than in terminal small-animal experiments [16, 65, 66, 92].

### Biomarker-specific and computational directions for clinically relevant EV imaging

A major unresolved challenge in the field is that most current imaging strategies detect labeled EV-associated signal without reliably distinguishing TEVs from the broader background of host-derived vesicles in circulation and tissues [65, 66, 96]. This limitation is particularly important in the context of premetastatic niche imaging, where signal enrichment in organs such as the lung, liver, or bone may reflect nonspecific biodistribution, reticuloendothelial sequestration, or label persistence rather than selective visualization of biologically active tumor-derived EVs [39, 65, 66]. Accordingly, future progress will depend not only on improved imaging sensitivity, but also on greater biological specificity [4, 96, 98].

One potential route toward greater specificity is the development of imaging strategies linked to EV-associated biomarkers or niche-associated molecular changes [96, 98, 99]. Candidate targets may include surface proteins such as PD-L1, organotropism-related adhesion molecules, and selected EV-associated nucleic acid cargoes, as well as downstream tissue markers associated with premetastatic niche formation such as inflammatory or stromal activation programs [15, 96, 99–102]. However, direct in-vivo visualization of such biomarkers remains technically difficult [65, 66]. In most current systems, imaging still relies on bulk vesicle labelling, engineered reporters, or contrast-loading approaches rather than direct detection of a defined tumor-EV subpopulation in its native state [16, 65, 66, 96]. Thus, biomarker-guided EV imaging should presently be viewed as an important research direction rather than an established clinical strategy [16, 65, 66, 96, 99].

Computational analysis may further strengthen this area by improving interpretation of EV-informed imaging data rather than replacing biological validation [65, 66, 98]. Artificial intelligence-based methods could support de-noising, segmentation, and quantitative feature extraction from CT, MRI, PET, and multimodal datasets, and may eventually help identify subtle tissue patterns associated with early niche remodeling [98, 100, 103]. More broadly, radiomics-style approaches may assist in integrating anatomical, functional, and molecular imaging signals with EV-associated biomarker information [98–100]. However, the principal bottleneck remains the limited availability of biologically validated datasets linking imaging features to TEV trafficking, stromal reprogramming, or biomarker-defined EV populations. [65, 66, 96] For this reason, artificial intelligence should currently be considered a complementary analytical layer that may enhance interpretation and hypothesis generation, but not a stand-alone solution for premetastatic niche detection [65, 66, 98].

Taken together, clinically relevant EV imaging will likely require convergence of three advances: improved tumor-EV specificity, imaging-compatible biomarker strategies, and computational frameworks capable of integrating multimodal signals without losing biological interpretability [65, 66, 96, 98, 100]. Until those elements are established in rigorously validated preclinical and translational studies, claims of direct EV-based premetastatic niche imaging should remain cautious [16, 65, 66].

## CONCLUSIONS AND PERSPECTIVES

Tumor-derived extracellular vesicles have emerged as important mediators of metastatic progression and plausible contributors to premetastatic niche formation through their effects on stromal, vascular, immune, and extracellular matrix remodeling [5, 10, 15, 17, 18, 40]. Their organ-targeted trafficking and capacity to carry endogenous or engineered imaging cargo make them attractive candidates for studying early metastatic conditioning and for developing biologically informed imaging strategies [15, 16, 60, 61].

At present, however, EV-based imaging remains predominantly a preclinical research tool (summarized in Figure 6). Most available studies visualize labeled or engineered EV-associated signals rather than the premetastatic niche directly, and the resulting readouts should therefore be interpreted as indirect indicators of tissue conditioning rather than clinically validated methods for early metastasis detection [16, 65, 66].

**Figure 6:**
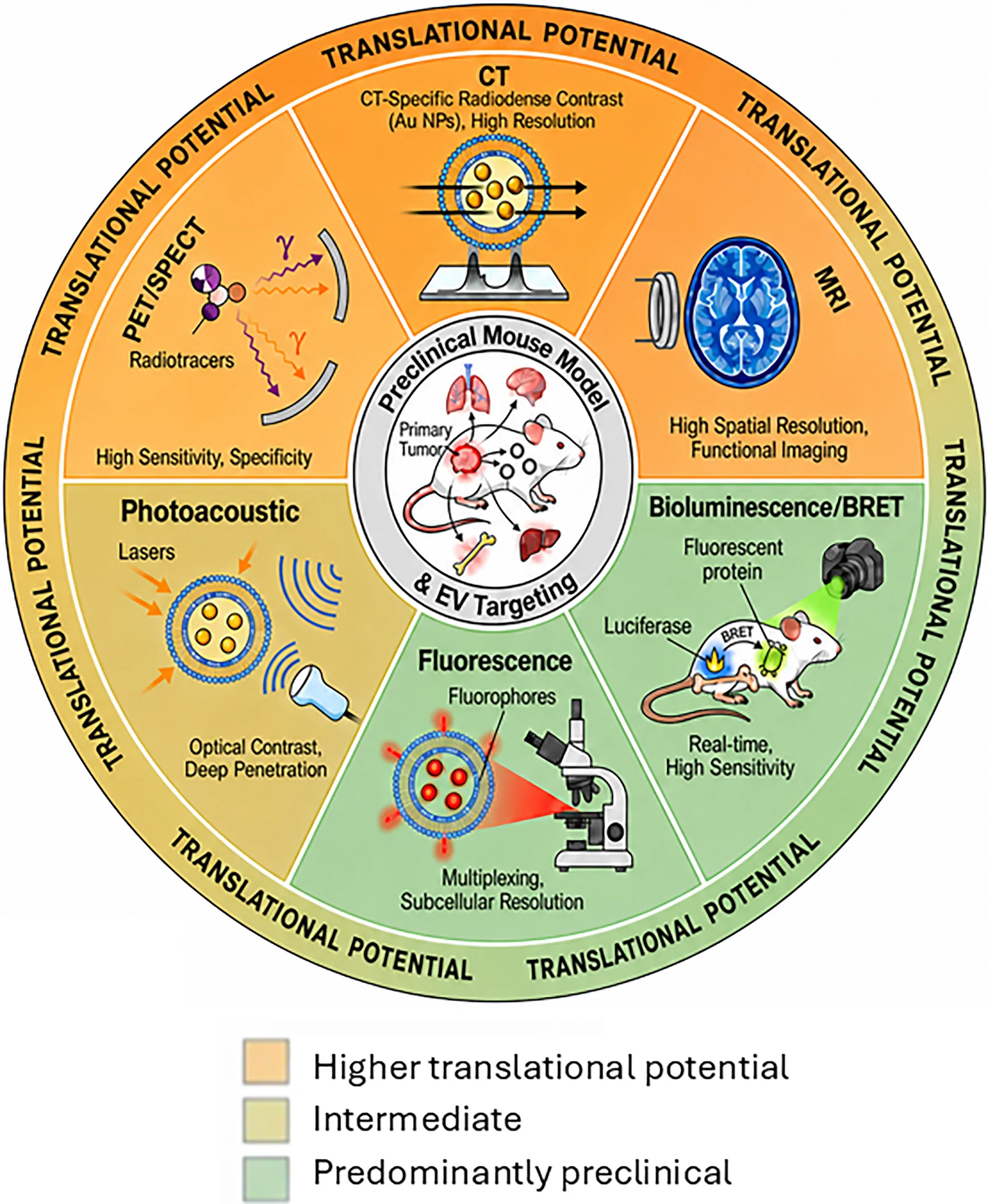
Translational feasibility of the EV tracking-based premetastatic niche detection. Tumor derived extracellular vesicles can be labelled or engineered using various multimodal contrast agents as illustrated in the schematic including radiolabelling (PET/CT) fluorescence labelling (FLI), photoacoustic imaging (PAI), magnetic resonance imaging (MRI) and bioluminescent labelling (IVIS). Feasibility scale: *Low*, predominantly restricted to preclinical investigation; *Medium*, translationally relevant but limited by substantial technical or regulatory barriers; *High*, comparatively most compatible with near-term clinical evaluation, although not yet clinically established for direct premetastatic niche imaging.

The most important next step is to improve biological fidelity, tumor specificity, and translational feasibility in parallel. Overall, EV-based imaging is best viewed as a promising but still evolving strategy whose future clinical value will depend on whether it can move beyond proof-of-concept biodistribution studies toward robust, tumor-specific, and clinically feasible approaches for premetastatic niches detections and early metastatic risk assessment [1, 65, 66, 92].

## SUPPLEMENTARY MATERIALS


