# Supplementary material for: Emerging biomedical imaging applications of tumor-derived extracellular vesicles for premetastatic niche detection: Biological rationale, engineering strategies, and translational challenges
Source: Oncotarget. 2026 Aug 28;17:389–412. doi: 10.18632/oncotarget.28916 (PMC13637956; doi:10.18632/oncotarget.28916)
Supplement: Supplementary file 1 [file oncotarget-17-28916-s001.pdf]

Emerging biomedical imaging applications of tumor-derived extracellular vesicles for premetastatic niche detection: Biological rationale, engineering strategies, and translational challenges

SUPPLEMENTARY MATERIALS

Supplementary Table 1: Detailed study-level inventory of representative extracellular vesicle (EV) imaging reports discussed in this review, including bibliography number, study/model, imaging probe or signal source, EV labeling strategy, *in vitro* validation method, *in vivo* imaging modality, and mode of probe association

Ref	Study/Model	Imaging probe or signal source	EV labeling strategy	*In vitro* validation method	*In vivo* imaging modality	Mode of probe association
[1]	SPECT imaging in BALB/c mice	Radioisotope (99mTc)	External	iTLC	SPECT/CT	Labeling
[2]	SPECT imaging in BALB/c mice	Radioisotope (99mTc)	External	Gamma counter	SPECT/CT	Labeling
[3]	CT imaging in a myocardial infarction mouse model	Gold nanoparticles	External	Not reported	CT	Incorporation
[4]	PET/CT imaging in C57BL/6J mice	Radioisotope ([89Zr] Zr(oxinate)4)	Internal	iTLC	PET/CT	Incorporation
[5]	PET/CT imaging in ICR mice and SD rats	Radioisotope (89Zr); fluorescent moiety (DFO)	External	Gamma counter	PET/CT	Labeling
[6]	PET/CT imaging in a subcutaneous 4T1 breast cancer model	Radioisotope (64Cu-NOTA-PEG)	External	Confocal microscopy	PET	Labeling
[7]	PET/CT imaging in a 22RV1 murine xenograft model	Radioisotope (124I/125I)	External	Not reported	Micro-PET/CT	Labeling
[8]	PET/CT imaging in a B16F10 murine xenograft model	Gold nanoparticles; fluorescent moiety (DiD CD63-RFP)	Internal	Confocal microscopy; flow cytometry	nanoSPECT/CT	Incorporation and expression
[9]	SPECT/CT imaging in 4T1 and AT3 xenograft models	Radioisotope (131I)	External	Not reported	SPECT/CT	Labeling
[10]	MPI/CT imaging in a 4T1BR5-L2G xenograft model	Superparamagnetic iron oxide (SPIO)	Internal	Confocal microscopy	MPI/CT	Incorporation
[11]	MRI imaging in C57BL/6 mice	Paramagnetic protein (FTH1)	External	Not reported	MRI	Expression
[12]	MRI/PET imaging in a 143B-Luc+ murine xenograft model	Radioisotope (NODAGA-64Cu); fluorescent moiety (ICG)	External	Confocal microscopy	PET/MRI	Labeling
[13]	MPI and IVIS imaging in a mouse prostate cancer model	Radioisotope and superparamagnetic moiety (DSPE-PEG-Cy7/SPION)	External and internal	Confocal microscopy	Optical imaging/MPI	Labeling and incorporation
[14]	IVIS and PET imaging in BALB/c nu/nu mice	Radioisotope and fluorescent moiety (Cy7-NOTA-64Cu/68Ga)	External	Confocal microscopy	Optical imaging/PET	Labeling
[15]	Fluorescent, bioluminescent, and radioactive imaging in CT26 tumor-bearing BALB/c mice	Radioisotope ([111In]-DTPA); fluorescent moiety (mCherry); bioluminescent moiety (Fluc/Nluc)	External/ internal	Flow cytometry	SPECT/CT/BLI	Labeling and expression
[16]	Nude mouse pancreatic cancer model, derived *in vitro* imaging	Fluorescent protein (pCT-CD63-GFP)	External	Confocal microscopy	Not reported	Expression

[17]	IVIS imaging in 4T1 and CT26 murine xenograft models	Fluorescent moiety (DiR/ DVDMS)	External/ internal	Confocal microscopy	Optical imaging	Labeling and incorporation
[18]	IVIS imaging in a B16F10 murine tumor model	Fluorescent moiety (ASL-BODIPY-PEG-cyclic RGD peptide)	External	Confocal microscopy	Optical imaging	Labeling
[19]	Non-invasive NIR-II fluorescence imaging in a U87 murine xenograft model	Fluorescent moiety (DiI-NIR-II C12)	External/ internal	Confocal microscopy	Optical imaging	Incorporation
[20]	Non-invasive NIR-II fluorescence imaging via biorthogonal click chemistry in a murine A549 xenograft model	Fluorescent moiety (DBCO-Cy5)	Internal	Confocal microscopy	Optical imaging	Incorporation
[21]	Ex vivo bioimaging of ICG-EXO with chicken breast tissues	Fluorescent moiety (ICG)	Internal	Confocal microscopy	Not reported	Incorporation
[22]	IVIS imaging in a peritonitis mouse model	Fluorescent moiety (BDP and SCy5)	External	Confocal microscopy; flow cytometry	Optical imaging	Labeling
[23]	IVIS imaging in CD1 mice	Fluorescent moiety (BDP and SCy7.5)	External	Confocal microscopy	Optical imaging	Labeling
[24]	*In vitro* fluorescence imaging of murine xenograft-derived RFP-MiaPaCa-2 and AsPC-1/ GFP cells	Fluorescent protein (pCT-CD63-GFP)	External	Confocal microscopy	Not reported	Expression
[25]	IVIS imaging in murine 4T1-BGL xenograft models	Bioluminescent moiety (PalmReNL)	Internal	Confocal microscopy; flow cytometry	Optical imaging	Expression
[26]	IVIS imaging in C57BL/6J and NMRI mice	Bioluminescent moiety (CD9/CD63-ThermoLuc)	External	Luminometer	Optical imaging	Expression
[27]	*In vitro* fluorescence imaging of L02 and HeLa cells	Fluorescent nanoparticle (DNA-Ag-Dox)	Internal	Confocal microscopy	Not reported	Incorporation
[28]	Fluorescence imaging of plasma membrane glycoprotein MUC1 and cytoplasmic miR-21 in MCF-7, HepG2, and L-02 cells	Fluorescent nanoparticle (fluorophore-labeled DNA aptamer)	External	Confocal microscopy	Not reported	Labeling
[29]	IVIS, MRI, and CT imaging in murine HeLa xenograft models	Fluorescent/paramagnetic nanoparticle (Ir and Fe nanoclusters)	Internal	Confocal microscopy	MRI/CT/optical imaging	Incorporation
[30]	Fluorescence imaging of EVs in H460, HaCaT, and A549 cells	Anti-PD-L1 monoclonal antibody-conjugated magnetic nanoparticle-attached yellow fluorescence carbon dot nanoarchitecture	External	Confocal microscopy	Not reported	Labeling
[31]	Fluorescence imaging in an *in vitro* model	Fluorescent nanoparticle (CsPbBr3 PQDs)	Internal	Single-photon luminescence imaging	Not reported	Incorporation
[32]	Photoacoustic imaging in an orthotopic U-87 brain tumor mouse model	Photoacoustic nanoparticle (Prussian blue-based nanoparticles)	External	Confocal microscopy	Photoacoustic imaging	Labeling
[33]	*In vitro* fluorescence detection of EV-bearing blood samples using anti-TNC antibody-conjugated YECD-CoFe2O4 nanoparticle-based nanoarchitecture	Fluorescent nanoparticle (CoFe2O4)	External	Time-resolved luminescence spectroscopy; SQUID magnetometry	Not reported	Labeling

[34]	Aptamer-based competitive fluorescence detection of EVs in complex biological samples	Fluorescent nanoparticle (aptamer-modified magnetic nanoparticles)	External	Not reported	Not reported	Labeling
[35]	Cre-loxP-based fluorescence imaging in an *in vitro*/*in vivo* system	Molecular biology reporter system (loxP-DsRed-stop-loxP-eGFP)	Internal	Confocal microscopy	Not reported	Incorporation
[36]	CRISPR-Cas9-based fluorescence imaging in an *in vitro*/*in vivo* system	Molecular biology reporter system (mCherryq2-linker-stop-eGFP)	Internal	Confocal microscopy	Not reported	Incorporation

Numbered entries in the Ref. column correspond to the Supplementary Table 1 bibliography. "Not reported" indicates that the relevant methodological detail was not clearly specified in the summarized source. Abbreviations: BLI: bioluminescence imaging; CT: computed tomography; EV: extracellular vesicle; iTLC: instant thin-layer chromatography; IVIS: *in vivo* imaging system; MPI: magnetic particle imaging; MRI: magnetic resonance imaging; PET: positron emission tomography; SPECT: single-photon emission computed tomography.

SUPPLEMENTARY REFERENCES

1. González MI, Martín-Duque P, Desco M, Salinas B. Radioactive Labeling of Milk-Derived Exosomes with ^{99m}Tc and In Vivo Tracking by SPECT Imaging. Nanomaterials (Basel). 2020; 10:1062. https://doi.org/10.3390/nano10061062. [PubMed]
2. Chung YH, Ho YP, Farn SS, Tsai WC, Li ZX, Lin TY, Weng CC. In vivo SPECT imaging of Tc-99m radiolabeled exosomes from human umbilical-cord derived mesenchymal stem cells in small animals. Biomed J. 2024; 47:100721. https://doi.org/10.1016/j.bj.2024.100721. [PubMed]
3. Gong L, Weng Y, Zhou W, Zhang K, Li W, Jiang J, Zhu J. *In vivo* CT imaging of gold nanoparticle-labeled exosomes in a myocardial infarction mouse model. Ann Transl Med. 2021; 9:504. https://doi.org/10.21037/atm-21-981. [PubMed]
4. Khan AA, Man F, Faruqu FN, Kim J, Al-Salemee F, Carrascal-Miniño A, et al. PET Imaging of Small Extracellular Vesicles via [89 Zr]Zr(oxinate) 4 Direct Radiolabeling. Bioconjugate Chemistry. 2022; 33:473–85.
5. Choi H, Kim MY, Kim DH, Yun H, Oh BK, Kim SB, Song IH, Park HS, Kim SE, Park C, Choi C. Quantitative Biodistribution and Pharmacokinetics Study of GMP-Grade Exosomes Labeled with ^{89}Zr Radioisotope in Mice and Rats. Pharmaceutics. 2022; 14:1118. https://doi.org/10.3390/pharmaceutics14061118. [PubMed]
6. Shi S, Li T, Wen X, Wu SY, Xiong C, Zhao J, Lincha VR, Chow DS, Liu Y, Sood AK, Li C. Copper-64 Labeled PEGylated Exosomes for In Vivo Positron Emission Tomography and Enhanced Tumor Retention. Bioconjug Chem. 2019; 30:2675–83. https://doi.org/10.1021/acs.bioconjchem.9b00587. [PubMed]
7. Guo Q, Zhao C, Gao X, Ding L, Wang P, Ren Y, Hou X, Yao Y, Zhang C, Yang X, Yang Z, Zhu H. One-Minute Iodine Isotope Labeling Technology Enables Noninvasive Tracking and Quantification of Extracellular Vesicles in Tumor Lesions and Intact Animals. Mol Pharm. 2023; 20:3672–82. https://doi.org/10.1021/acs.molpharmaceut.3c00299. [PubMed]
8. Lara P, Palma-Florez S, Salas-Huenuleo E, Polakovicova I, Guerrero S, Lobos-Gonzalez L, Campos A, Muñoz L, Jorquera-Cordero C, Varas-Godoy M, Cancino J, Arias E, Villegas J, et al. Gold nanoparticle based double-labeling of melanoma extracellular vesicles to determine the specificity of uptake by cells and preferential accumulation in small metastatic lung tumors. J Nanobiotechnology. 2020; 18:20. https://doi.org/10.1186/s12951-020-0573-0. [PubMed]
9. Rashid MH, Borin TF, Ara R, Angara K, Cai J, Achyut BR, Liu Y, Arbab AS. Differential *in vivo* biodistribution of ^{131}I-labeled exosomes from diverse cellular origins and its implication for theranostic application. Nanomedicine. 2019; 21:102072. https://doi.org/10.1016/j.nano.2019.102072. [PubMed]
10. Toomajian VA, Tundo A, Ural EE, Greeson EM, Contag CH, Makela AV. Magnetic Particle Imaging Reveals that Iron-Labeled Extracellular Vesicles Accumulate in Brains of Mice with Metastases. ACS Appl Mater Interfaces. 2024; 16:30860–73. https://doi.org/10.1021/acsami.4c04920. [PubMed]
11. Liu T, Li Z, Li X, Zhao R, Wei X, Wang Z, Xin SX. In vivo visualization of murine melanoma cells B16-derived exosomes through magnetic resonance imaging. Biochim Biophys Acta Gen Subj. 2022; 1866:130062. https://doi.org/10.1016/j.bbagen.2021.130062. [PubMed]
12. Almeida SFF, Fonseca A, Sereno J, Ferreira HRS, Lapo-Pais M, Martins-Marques T, Rodrigues T, Oliveira RC, Miranda C, Almeida LP, Girão H, Falcão A, Abrunhosa AJ, Gomes CM. Osteosarcoma-Derived Exosomes as Potential PET Imaging Nanocarriers for Lung Metastasis. Small. 2022; 18:e2203999. https://doi.org/10.1002/smll.202203999. [PubMed]
13. Liu S, Shang W, Song J, Li Q, Wang L. Integration of photomagnetic bimodal imaging to monitor an autogenous exosome loaded platform: unveiling strong targeted retention effects for guiding the photothermal and magnetothermal therapy in a mouse prostate cancer model. J Nanobiotechnology. 2024; 22:421. https://doi.org/10.1186/s12951-024-02704-0. [PubMed]
14. Jung KO, Kim YH, Chung SJ, Lee CH, Rhee S, Pratx G, Chung JK, Youn H. Identification of Lymphatic and Hematogenous Routes of Rapidly Labeled Radioactive and Fluorescent Exosomes through Highly Sensitive Multimodal Imaging. Int J Mol Sci. 2020; 21:7850. https://doi.org/10.3390/ijms21217850. [PubMed]

15. Lázaro-Ibáñez E, Faruqu FN, Saleh AF, Silva AM, Tzu-Wen Wang J, Rak J, Al-Jamal KT, Dekker N. Selection of Fluorescent, Bioluminescent, and Radioactive Tracers to Accurately Reflect Extracellular Vesicle Biodistribution *in Vivo*. ACS Nano. 2021; 15:3212–27. https://doi.org/10.1021/acsnano.0c09873. [PubMed]
16. Satake T, Suetsugu A, Nakamura M, Kunisada T, Saji S, Moriwaki H, Shimizu M, Hoffman RM. Color-coded Imaging of the Fate of Cancer-cell-derived Exosomes During Pancreatic Cancer Metastases in a Nude-mouse Model. Anticancer Res. 2019; 39:4055–60. https://doi.org/10.21873/anticanres.13561. [PubMed]
17. Liu Y, Bai L, Guo K, Jia Y, Zhang K, Liu Q, Wang P, Wang X. Focused ultrasound-augmented targeting delivery of nanosonosensitizers from homogenous exosomes for enhanced sonodynamic cancer therapy. Theranostics. 2019; 9:5261–81. https://doi.org/10.7150/thno.33183. [PubMed]
18. Kang C, Han P, Lee JS, Lee D, Kim D. Anchor, Spacer, and Ligand-Modified Engineered Exosomes for Trackable Targeted Therapy. Bioconjug Chem. 2020; 31:2541–52. https://doi.org/10.1021/acs.bioconjchem.0c00483. [PubMed]
19. Liu Y, Li M, Gu J, Huang H, Xie H, Yu C, Roy S, Chen X, Kuang T, Zhang Y, Jiang S, Guo B. Engineering of exosome-liposome hybrid-based theranostic nanomedicines for NIR-II fluorescence imaging-guided and targeted NIR-II photothermal therapy of subcutaneous glioblastoma. Colloids Surf B Biointerfaces. 2025; 245:114258. https://doi.org/10.1016/j.colsurfb.2024.114258. [PubMed]
20. Song S, Shim MK, Lim S, Moon Y, Yang S, Kim J, Hong Y, Yoon HY, Kim IS, Hwang KY, Kim K. In Situ One-Step Fluorescence Labeling Strategy of Exosomes via Bioorthogonal Click Chemistry for Real-Time Exosome Tracking In Vitro and In Vivo. Bioconjug Chem. 2020; 31:1562–74. https://doi.org/10.1021/acs.bioconjchem.0c00216. [PubMed]
21. Cotto NM, Chauhan N, Adriano B, Chauhan DS, Cabrera M, Chauhan SC, Yallapu MM. Milk Exosome-Glow Nanosystem for Cancer Cellular and Tissue Bioimaging. Chem Biomed Imaging. 2024; 2:711–20. https://doi.org/10.1021/cbmi.4c00040. [PubMed]
22. Santos-Coquillat A, González MI, Clemente-Moragón A, González-Arjona M, Albaladejo-García V, Peinado H, Muñoz J, Ximénez Embún P, Ibañez B, Oliver E, Desco M, Salinas B. Goat Milk Exosomes As Natural Nanoparticles for Detecting Inflammatory Processes By Optical Imaging. Small. 2022; 18:e2105421. https://doi.org/10.1002/smll.202105421. [PubMed]
23. González MI, González-Arjona M, Santos-Coquillat A, Vaquero J, Vázquez-Ogando E, de Molina A, Peinado H, Desco M, Salinas B. Covalently Labeled Fluorescent Exosomes for In Vitro and In Vivo Applications. Biomedicines. 2021; 9:81. https://doi.org/10.3390/biomedicines9010081. [PubMed]
24. Yamashita K, Suetsugu A, Hayashi S, Shimizu M, Hoffman RM. Exosome Transfer Between Different Co-implanted Human Pancreatic Cancer Cell Lines Occurs in the Primary Tumor and Multiple Metastatic Sites of Nude Mice But Not in Co-Culture *In Vitro*. Anticancer Res. 2024; 44:3307–15. https://doi.org/10.21873/anticanres.17149. [PubMed]
25. Perez GI, Broadbent D, Zarea AA, Dolgikh B, Bernard MP, Withrow A, McGill A, Toomajian V, Thampy LK, Harkema J, Walker JR, Kirkland TA, Bachmann MH, et al. In Vitro and In Vivo Analysis of Extracellular Vesicle-Mediated Metastasis Using a Bright, Red-Shifted Bioluminescent Reporter Protein. Adv Genet (Hoboken). 2022; 3:2100055. https://doi.org/10.1002/ggn2.202100055. [PubMed]
26. Gupta D, Liang X, Pavlova S, Wiklander OPB, Corso G, Zhao Y, Saher O, Bost J, Zickler AM, Piffko A, Maire CL, Ricklefs FL, Gustafsson O, et al. Quantification of extracellular vesicles *in vitro* and *in vivo* using sensitive bioluminescence imaging. J Extracell Vesicles. 2020; 9:1800222. https://doi.org/10.1080/20013078.2020.1800222. [PubMed]
27. Qambrani A, Rehman FU, Tanziela T, Shaikh S, Semcheddine F, Du T, Liu W, Jiang H, Wang X. Biocompatible exosomes nanodrug cargo for cancer cell bioimaging and drug delivery. Biomed Mater. 2021; 16:025026. https://doi.org/10.1088/1748-605X/abaaa2. [PubMed]
28. Chen ML, He F, Yin BC, Ye BC. Simultaneous imaging of cancer biomarkers in live cells based on DNA-engineered exosomes. Analyst. 2021; 146:1626–32. https://doi.org/10.1039/d0an02353a. [PubMed]
29. Shaikh S, Rehman FU, Du T, Jiang H, Yin L, Wang X, Chai R. Real-Time Multimodal Bioimaging of Cancer Cells and Exosomes through Biosynthesized Iridium and Iron Nanoclusters. ACS Appl Mater Interfaces. 2018; 10:26056–63. https://doi.org/10.1021/acsami.8b08975. [PubMed]
30. Pramanik A, Patibandla S, Gao Y, Corby LR, Rhaman MM, Sinha SS, Ray PC. Bio-Conjugated Magnetic-Fluorescence Nanoarchitectures for the Capture and Identification of Lung-Tumor-Derived Programmed Cell Death Lighand 1-Positive Exosomes. ACS Omega. 2022; 7:16035–42. https://doi.org/10.1021/acsomega.2c01210. [PubMed]
31. Pramanik A, Gates K, Patibandla S, Davis D, Begum S, Iftekhar R, Alamgir S, Paige S, Porter MM, Ray PC. Water-Soluble and Bright Luminescent Cesium-Lead-Bromide Perovskite Quantum Dot-Polymer Composites for Tumor-Derived Exosome Imaging. ACS Appl Bio Mater. 2019; 2:5872–79. https://doi.org/10.1021/acsabm.9b00837. [PubMed]
32. Hill ML, Chung SJ, Woo HJ, Park CR, Hadrick K, Nafiujjaman M, Kumar PPP, Mwangi L, Parikh R, Kim T. Exosome-Coated Prussian Blue Nanoparticles for Specific Targeting and Treatment of Glioblastoma. ACS Appl Mater Interfaces. 2024; 16:20286–301. https://doi.org/10.1021/acsami.4c02364. [PubMed]
33. Pramanik A, Kundu S, Gates K, Kumar A, Kolawole OP, Talukdar A, Iftekhar R, Corby LR, Ray PC. Multi-Color-Emissive Magneto-Luminescent Nanoarchitectures for Targeted Identification of Heterogeneous Exosomes Associated with Lung Cancer Metastasis. ACS Appl

Bio Mater. 2023; 6:2446–58. https://doi.org/10.1021/acsabm.3c00255. [PubMed]

34. Yu X, He L, Pentok M, Yang H, Yang Y, Li Z, He N, Deng Y, Li S, Liu T, Chen X, Luo H. An aptamer-based new method for competitive fluorescence detection of exosomes. Nanoscale. 2019; 11:15589–95. https://doi.org/10.1039/c9nr04050a. [PubMed]

35. Zomer A, Steenbeek SC, Maynard C, van Rheenen J. Studying extracellular vesicle transfer by a Cre-loxP method. Nat Protoc. 2016; 11:87–101. https://doi.org/10.1038/nprot.2015.138. [PubMed]

36. de Jong OG, Murphy DE, Mäger I, Willms E, Garcia-Guerra A, Gitz-Francois JJ, Lefferts J, Gupta D, Steenbeek SC, van Rheenen J, El Andaloussi S, Schiffelers RM, Wood MJA, Vader P. A CRISPR-Cas9-based reporter system for single-cell detection of extracellular vesicle-mediated functional transfer of RNA. Nat Commun. 2020; 11:1113. https://doi.org/10.1038/s41467-020-14977-8. [PubMed]
